# Cognitive implications of rheumatoid arthritis: A call for comprehensive care and research focus

**DOI:** 10.1002/iid3.1065

**Published:** 2023-11-07

**Authors:** Marium Qidwai, Khadija Ahmed, Muhammad Fawad Tahir, Sean Kaisser Shaeen, Muhammad Hasanain, Abdullah Malikzai

**Affiliations:** ^1^ Department of Medicine Dow Medical College Karachi Pakistan; ^2^ Department of Medicine and Surgery HBS Medical and Dental College Islamabad Pakistan; ^3^ Kabul University of Medical Sciences Kabul Afghanistan

**Keywords:** autoimmune disease, awareness, cognitive impairment, inflammation, patient care team, rheumatoid arthritis, treatment outcome

## 1 INTRODUCTION/BACKGROUND

Rheumatoid arthritis (RA) is an autoimmune disorder that primarily manifests as symmetrical joint pain, swelling, stiffness, and fatigue. It is the most common inflammatory arthritis where cardinal joint symptoms emerge due to inflammation of the articular surfaces of the joints and their surrounding soft tissues. Persistent inflammation results in structural changes of the joints and consequently impaired physical function. It is a chronic, crippling disorder that typically develops between the ages of 20 and 40 and is more common among women.^1^ RA is a multisystem disorder that frequently presents with extra‐articular manifestations, including respiratory infections, cardiovascular disease, neurological problems, and vasculitis and so on.^2^ Although RA rarely exhibits neurological symptoms, the impact of cognitive impairment in RA, according to recent research, may be substantial.^3^ Cognitive Impairment is the slow deterioration of mental capacities like learning, remembering, paying attention, reasoning, and decision‐making. Multiple factors have been identified as potential contributors of cognitive impairment in RA patients, encompassing autoimmune and inflammatory components, cardiovascular complications, psychiatric disorders, persistent pain, medication side effects, age, genetic factors, and hormonal fluctuations.^4^

Premature aging of the immune system, the presence of autoantibodies, and pro‐inflammatory substances have been implicated in the development of cognitive impairment in RA patients.^5^ Chronic inflammation may play a role in the development of depression in RA patients. This can be attributed to the immune‐mediated inflammatory response seen in RA that affects brain cells. Depression as a result may ultimately lower the quality of life.^6^ Cardiovascular complications like stroke and heart attack are common in RA patients and may contribute to cognitive impairment due to the underlying inflammation of the vessels. The cerebrovascular dysfunction suggests a potential connection between dementia and RA.^4^ Chronic pain is another factor that influences cognitive function in RA patients.^1^ Given the strong connections between the brain systems involved in cognition and pain perception, it is hypothesized that these systems could affect one another.^7^ The treatment of RA that mainly comprises immunosuppressives are known to cause neurologic dysfunction when used for longer periods of time.^8^ Methotrexate, although highly efficacious, carries a significant risk of cognitive impairment, confusion, and mood changes.^9^ Additionally, age plays a role, with cognitive impairment being more evident in older patients.^10^ Elderly individuals with long‐standing RA have a higher risk of accelerated inflammatory atherosclerosis and stroke hence increasing their susceptibility to cognitive impairment.^4^ However, cognitive impairment has also been observed in younger RA patients, especially during the early stages of the disease.^4^

Cognitive impairment in RA patients is a worldwide concern. In the United Kingdom, there is significant concern about cognitive impairment among individuals with RA, as evidenced by a recent study that employed the Montreal Cognitive Assessment (MoCA) tool to assess cognitive impairment in older RA patients.^11^ This study revealed that a substantial portion, 72.2%, of the participants were classified as cognitively impaired based on a MoCA cutoff score of ≤27/30 points.

These findings parallel research conducted in various other countries, including Italy and South Korea, where similar high prevalences of cognitive impairment in RA patients have been documented. For instance, Bartolini et al.^12^ reported cognitive impairment in the range of 38%–71% in their RA cohort, with particular difficulties observed in visuospatial/executive function tasks. Similarly, Shin et al.^13^ identified cognitive impairment rates varying from 8% to 29%, depending on the specific cognitive domain assessed. Appenzeller et al.^14^ found that 30% of their RA cohort exhibited cognitive impairment as measured through various neuropsychological assessments. These collective findings underscore the substantial cognitive burden experienced by RA patients across different countries.

The purpose of this commentary is to shed light on the often‐overlooked aspect of cognitive impairment in RA. It aims to emphasize the significant impact of cognitive decline on the lives of RA patients and the challenges it poses for both patients and medical professionals. The commentary also highlights efforts to address this issue, including education, multidisciplinary care, and recommendations from organizations like the Centers for Disease Control and Prevention (CDC). It calls for increased awareness, proactive approaches to diagnosis and management, and further research to improve the understanding and treatment of cognitive impairment in individuals with RA.

## 2 CHALLENGES

Cognitive impairment in RA poses considerable challenges for both patients and medical professionals. Since cognitive impairment is not a typical presentation of RA, it often goes unnoticed. This lack of awareness often leads to late diagnosis, hindrance in provision of prompt therapies and timely care of the patients, hence resulting in poor prognosis.

The issues are further compounded by the difficulty in identifying cognitive impairment in RA patients. Memory loss, concentration problems, and executive dysfunction are examples of cognitive impairment symptoms that can coexist with other RA symptoms or perhaps be explained by aging‐related changes. This makes it difficult to distinguish between normal aging, cognitive impairment brought on by RA, and other possible reasons, which results in underdiagnosis and insufficient care.

The scarcity of research that has been done on cognitive impairment in RA presents another difficulty. There is still a lack of knowledge of the underlying processes, risk factors, and ideal treatment approaches for cognitive impairment in RA. The lack of publications on this particular topic makes it difficult to establish therapies and evidence‐based guidelines that are especially suited to addressing cognitive impairment in RA patients.

Additionally, there is a dearth of funding for research on cognitive impairment in RA. To undertake thorough investigations, including neuroimaging, neuropsychological evaluations, and longitudinal follow‐up, research in this area needs financial resources. The lack of financing makes it difficult to learn more about cognitive impairment and how it affects RA patients, which hinders efforts to improve patient outcomes.

A major issue is also posed by patients' noncompliance with their RA treatment plan.^15^ The capacity of patients to take their medications as prescribed, follow dietary advice, and go to scheduled follow‐up appointments may be compromised by cognitive impairment. This noncompliance might intensify illness symptoms and worsen cognitive impairment in RA patients.

These problems demand multifaceted approaches to solve. It is critical to raise patient and healthcare provider awareness of the possible cognitive effects of RA.

## 3 EFFORTS

A comprehensive plan that integrates a combination of strategies that addresses psychological issues in conjunction with medical treatment are being applied. Educating patients and using cognitive‐behavioral therapy is an effective psychological treatment for chronic pain diseases like RA.^11^ Such intervention programs have exhibited substantial advantages in diminishing disability, enhancing cognitive function, and promoting mental well‐being.^1^

Given the established connection between cognitive impairment in RA patients and depression and anxiety, it is evident that stress can further worsen cognitive impairment in individuals with RA.^16^ Therefore, regular exercise is strongly advised by physicians to mitigate these effects and can benefit patients with RA by exerting its psychological impact and may have an impact on depression symptoms.^16^ Techniques such as meditation, yoga, and deep breathing exercises have shown to be helpful in reducing stress and therefore improving cognitive function.^1^ Thus, yoga is a valuable adjunct therapy for individuals with RA and is recommended by doctors to RA patients to reduce the risk of cognitive impairment.^1^

Additional lifestyle changes including dietary modifications can also be integrated in this context as doctors recommend a healthy diet rich in fruits, vegetables, whole grains, and lean protein due to their benefits in helping the reduction of inflammation and, therefore, ameliorating cognitive function in patients with RA^10^, ^17^ Likewise, Omega‐3 fatty acids that are found in fatty fish and fish oil supplements, have further exhibited anti‐inflammatory attributes and might positively impact cognitive function.^12^ Furthermore, according to the WHO, by reducing or completely halting the consumption of alcohol and the cessation of tobacco, the risk of cognitive impairment can be mitigated even more so.^13^ Few research have been done to explore the underlying mechanism, risk factors and presentation of cognitive impairment in RA by researchers in the last few years. Although attempts have been made to shed light on this subject, there is still much that needs to be researched to improve our understanding and identify pharmacological interventions and effective management strategies.

Cognitive impairment has been documented in several other rheumatic diseases, including conditions such as gout,^18^ fibromyalgia,^19^ and osteoarthritis.^20^ Efforts to reduce cognitive impairment in various rheumatoid disorders, including RA, share common goals of improving the quality of life for affected individuals. As such, strategies to mitigate cognitive impairment may vary depending on the underlying disorder. Nonetheless, across these conditions, there is a growing recognition of the need for holistic care, including lifestyle modifications, medication management, and patient education, to address cognitive health alongside the primary disease management. Research and clinical efforts continue to explore the unique challenges and effective interventions for each rheumatoid disorder to enhance cognitive well‐being.

## 4 RECOMMENDATIONS

As a community, we need to raise awareness about cognitive impairment in RA. We need to ensure that cognitive impairment is recognized as a valid and significant aspect of RA, and that patients receive the timely comprehensive care they deserve. Efficient measures should be taken on both the individual and societal levels to address this issue effectively. This can be done by first conducting widespread educational campaigns that provide accurate information about how cognitive impairment can manifest in patients with RA and which notable symptoms should prompt patients and their attendants to seek immediate medical care. Second, the medical community should also acknowledge the increased need for research in this domain. Insufficient scholarly articles exist on this particular topic, thus necessitating further studies to acquire comprehensive understanding of this issue. Public events like seminars, health fairs and workshops can be organized to disseminate information about the disease. At these events, healthcare professionals can explain the association between RA and cognitive impairment and people affected by this condition can also share their personal experiences. This will lead to open discussions about this condition. Moreover, online platforms, media engagement and support groups can be taken into consideration to share information, patient experiences, treatment advancements, and research breakthroughs. Furthermore, physicians should incorporate regular cognitive screening as part of the routine care for patients with RA. This will enable the early detection and monitoring of cognitive impairment in individuals with RA, facilitating timely interventions and support. Multidisciplinary care should be implemented where a team of doctors including rheumatologists, neurologists, and psychiatrists should be involved in patient care. Efforts should be made by healthcare organizations worldwide to develop clinical guidance systems for such patients to enhance the disease outcome.

CDC also recommends several low‐cost strategies to enhance the quality of life for individuals with RA.^18^ These include getting physically active, with a target of 150 min of moderate activity weekly; attending effective physical activity programs for pain reduction and improved mobility; joining self‐management education classes to control symptoms and adapt to life with arthritis; quitting smoking to mitigate disease worsening; and maintaining a healthy weight to manage RA‐related challenges. These evidence‐based recommendations can significantly benefit those living with RA.

## 5 CONCLUSION

It is time to shine a spotlight on this often‐overlooked aspect of the disease and work toward better understanding the detection of and management of cognitive impairment in individuals living with RA. Efforts are being made in this regard through research, pharmacological, lifestyle, and nutritional interventions. However, we still need to develop a more proactive approach for early diagnosis and effective management of this disease. It is imperative to spread awareness about this often‐overlooked aspect of RA and healthcare providers should remain vigilant. More research should be done and multidisciplinary care of the patients should be carried out. These active steps can make a difference in the lives of those affected by this chronic condition.

## AUTHOR CONTRIBUTIONS

Marium Qidwai, Khadija Ahmed, and Sean Kaisser Shaeen contributed to the conception of this study. Marium Qidwai, Khadija Ahmed, and Muhammad Fawad Tahir did the drafting of the work. Sean Kaisser Shaeen critically revised the manuscript. Muhammad Hasanain and Sean Kaisser Shaeen reviewed the manuscript. Sean Kaisser Shaeen, Muhammad Hasanain, and Abdullah Malikzai gave the final approval and agreed to the accuracy of the work. All authors have read and agreed to the final version of the manuscript.

## CONFLICT OF INTEREST STATEMENT

The authors declare no conflict of interest.

## ETHICS STATEMENT

No humans or animal subjects were involved in this research.

## Data Availability

All data are included in the manuscript.
